# Cryo-EM structure of the hyperpolarization-activated inwardly rectifying potassium channel KAT1 from Arabidopsis

**DOI:** 10.1038/s41422-020-00407-3

**Published:** 2020-09-08

**Authors:** Siyu Li, Fan Yang, Demeng Sun, Yong Zhang, Mengge Zhang, Sanling Liu, Peng Zhou, Chaowei Shi, Longhua Zhang, Changlin Tian

**Affiliations:** 1 Hefei National Laboratory of Physical Sciences at Microscale, Anhui Laboratory of Advanced Photonic Science and Technology and School of Life Sciences, University of Science and Technology of China, Hefei, Anhui 230026 China; 2 High Magnetic Field Laboratory, Chinese Academy of Sciences, Hefei, Anhui 230030 China

**Keywords:** Cryoelectron microscopy, Plant signalling

Dear Editor,

Plants utilize K^+^ ions to maintain hydrostatic pressure, drive irreversible cell expansion for growth, and facilitate reversible changes in guard cell volume that cause stomatal opening or closing. KAT1 is a voltage-dependent potassium channel from *Arabidopsis thaliana* that is mainly expressed in guard cells. KAT1 allows the influx of K^+^, leading to the swelling and opening of the stoma, and therefore plays a key role in regulating the aperture of stomatal pores on the surface of plant leaves.^1–3^ To understand the gating mechanism of plant K^+^ channels poses several challenges, despite many structural similarities between these plant K^+^ channels and mammalian Kv and Shaker channels.^4^ Remarkably, most voltage-gated ion channels, such as Na^+^ (Nav), Ca^2+^ (Cav), and K^+^ (Kv) channels, open when the cell membrane is depolarized (when the voltage is positive inside relative to outside). Comparing with conventional depolarized K^+^ channels, KAT1 has a uniquely reversed voltage dependence: depolarization causes closing, and hyperpolarization causes opening.^3^ KAT1 thus falls into a rare class of hyperpolarization-activated channels, which include hyperpolarization-activated, cyclic nucleotide-gated (HCN) channels in animals, and KAT and AKT channels in plants.

Mechanistic studies have focused predominantly on the depolarization-activated ion channels. The mechanism underlying the voltage sensor control of the gate in hyperpolarization-activated ion channels is little studied. To date, the only resolved structure of a hyperpolarization-activated channel is that of HCN1.^5,6^ The cryo-EM structure of the HCN1 channel in a “hyperpolarized” state reveals that the long S4 helix breaks into two helices, with one running parallel to the membrane surface, analogous to the S4–S5 linker of domain-swapped voltage-gated channels. These findings suggest a basis for allosteric communication between voltage sensors and the gate in hyperpolarization-gated ion channels. However, preliminary sequence analysis shows that KAT1 and HCN1 have low sequence similarity. The S4 helix of KAT1 is much shorter than that of HCN1. This leads to the question of whether the structure and proposed gating mechanism of HCN1 can fully recapitulate those of KAT1. The intriguing biophysical properties of KAT1 motivate us to elucidate its molecular architecture.

Full-length KAT1 from Arabidopsis was cloned and transfected into Sf9 cells for expression. However, we failed to obtain KAT1 protein from the membrane fraction. Then we co-expressed KAT1 with KAB1, a structural component of some plant K^+^ channels,^7^ and eventually obtained highly stable and homogeneous KAT1 proteins uniform in composition as indicated by gel filtration and SDS-PAGE analysis (Supplementary information, Fig. S1). Unexpectedly, the corresponding band of KAB1 was not observed in the purification gel. This indicates that KAB1 could not form complex with KAT1 in vitro, but rather acts as a chaperon that facilitates KAT1 translocation to the membrane in Sf9 cells. The purified KAT1 was subjected to cryo-EM studies. A three-dimensional EM map was reconstructed to an overall resolution of 3.2 Å (Supplementary information, Figs. S2 and S3). The secondary-structure features of KAT1, particularly those of the majority of α-helices within the transmembrane layers, were clearly demonstrated (Supplementary information, Figs. S3 and S4). Finally, an atomic model of the structured core of KAT1 (residues 49–492) was readily built (Supplementary information, Table S1). Cryo-EM structure shows that KAT1 assembles as a tetramer channel with a 4-fold symmetry (Fig. 1a). Each subunit contains a voltage sensor domain (VSD, helices S1–S4) and a pore-forming region (helices S5–S6) inside the membrane bilayer (Fig. 1b). The S6 helix makes a sharp bend at its C terminal and gives rise to a helix–turn–helix motif named the “C-linker”. Following the C-linker, the polypeptide chain gives rise to five additional short α-helices and a β-jelly roll forming the cyclic nucleotide-binding domain (CNBD) (Fig. 1b). When this manuscript was being prepared, Michael et al.^8^ reported the cryo-EM structure of a functional construct of KAT1 (KAT1em) at 3.8 Å resolution. The structure shows that the truncated KAT1 assembles as an unusual dimer of two tetrameric channels stacking via their cytoplasmic domains. Detailed structural comparison shows that the overall architectures of the full-length KAT1 (reported here) and KAT1em tetramer are almost the same, and the structures of the single subunits of KAT1 and KAT1em are identical (Supplementary information, Fig. S5).

**Fig. 1:**
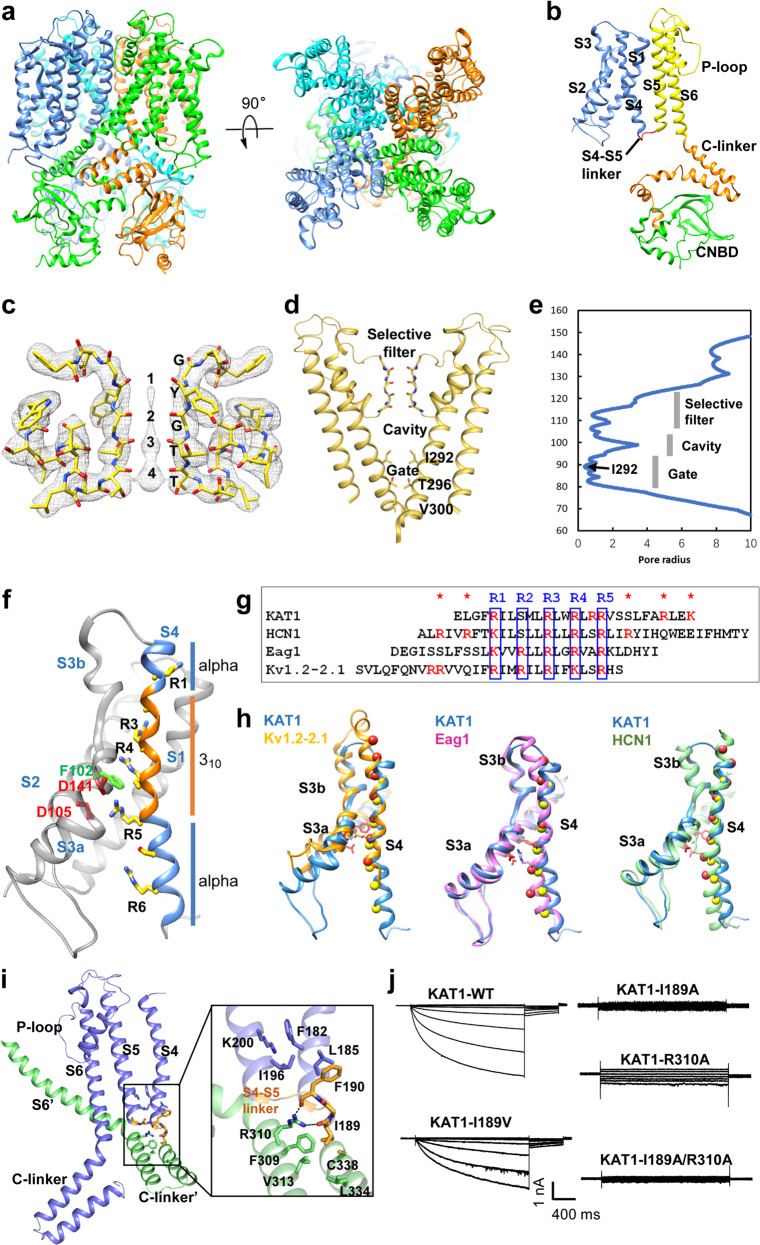
Cryo-EM structure of Arabidopsis KAT1 channel. **a** Side and top views of the cryo-EM structure of the KAT1 channel. The four subunits are shown in different colors. **b** Structure of a single subunit of the KAT1 channel showing a non-domain-swapped structure. The VSD (S1–S4), pore domain (S5–S6), C-linker and CNBD are colored blue, yellow, orange and green, respectively. The short non-helical S4–S5 linker is colored red. **c** Selectivity filter of KAT1 with densities in the pore from the ~3.2 Å map. The four potential K^+^ ion-binding sites (1 to 4) are labeled. **d** Structure of the pore. The ion pore of KAT1 is shown with only two subunits, viewed from the membrane. **e** The pore radius along the ion conduction pathway. **f** Structure of the VSD. Positions of Arg165 (R1), Ser168, Arg171 (R3), Arg174 (R4), Arg177 (R5), Ser180, and Arg184 (R6) are indicated. Residues forming the gating charge transfer center (Phe102, Asp105, Asp141) are shown as sticks. **g** Sequence alignment of the S4 helices from KAT1, HCN1, Eag1 and Kv1.2-2.1. Positively charged residues are highlighted. **h** Superposition of the VSD of KAT1 and those of Kv1.2-2.1 (PDB: 2R9R), Eag1 (PDB: 5K7L) and HCN1 (PDB: 5U6O). The Cα positions of the charged residues in KAT1 are shown as yellow spheres. The Cα positions of the charged residues in Kv1.2-2.1, Eag1 and HCN1 are shown as red spheres. **i** Interactions between the VSD, pore, and cytoplasmic domains. Close view of the possible interactions between residues in S4 and S5 helices, and residues in the S4-S5 linker and the C-linker are shown. **j** Patch-clamp electrophysiology analysis of wild-type KAT1 (KAT1-WT) and KAT1 mutants: KAT1-I189V, I189A, R310A, I189A/R310A.

An important first conclusion is that KAT1 is a non-domain-swapped ion channel. Each VSD of KAT1 is contiguous with the pore domain from the same subunit, and connected to the pore domain through a short, non-helical turn (named S4–S5 linker) (Fig. 1b). The non-helical S4–S5 linker in KAT1 is similar to those of other non-domain-swapped ion channels such as Eag1 (Kv10.1)^9^ and hERG (Kv11.1),^10^ but unlike those of domain-swapped ion channels such as Kv1.2-2.1^11^ (Supplementary information, Fig. S6). Moreover, the overall architecture of KAT1 superposes well with those of Eag1 and hERG, highlighting a high structural similarity between KAT1 and the two channels (Supplementary information, Fig. S7). Therefore, KAT1 is topologically similar to Kv10-12 channels, but unlike Shaker (Kv1-7) channels.

The selectivity filter of KAT1 is formed by four copies of P-loop, which is composed of Thr261–Thr262–Gly263–Tyr264–Gly265, an example of the universal TxGYG motif in the majority of potassium-selective channels (Fig. 1c). The carbonyl oxygen atoms of Thr261–Thr262–Gly263–Tyr264 in all four P-loops face inward to form coordination sites for K^+^ ions. The density map shows that four ion-binding sites exist within the selectivity filter (Fig. 1c). The structure of the selectivity filter in KAT1 is almost identical to those of Kv1.2-2.1 and Kir3.1, with root-mean-square deviations (RMSDs) of 0.32 Å and 0.29 Å for Cα atoms, respectively (Supplementary information, Fig. S8). Under the selectivity filter, S6 helices pack tightly to form the channel pore and the gate. The pore domain displays a closed inner gate, with its narrowest constriction formed by the hydrophobic side chains of Ile292 (Fig. 1d, e). The conductive conformation of the selectivity filter and the closed gate correspond to the expected closed state of KAT1 at 0 mV.

In voltage-gated ion channels, the S4 helix is the structural element that senses the membrane electric field. The structure of KAT1 shows that the middle of the S4 helix adopts a 3_10_ conformation, whereas the upper and lower parts are α-helices (Fig. 1f). The 3_10_-helix is comprised of residues (**R**IL)(**S**ML)(**R**LW)(**R**LR)(**R**VS) (Fig. 1g), but not of the strict triplets (**R/K**xx)_n_, which compose the VSD in the majority of voltage-gated channels. The second arginine is replaced by ser ine, which is rarely observed in voltage-gated channels. KAT1 has a gating charge transfer center^12^ formed by Phe102 and Asp105 from the S2 helix and Asp141 from the S3 helix (Fig. 1f). R5 (Arg177) interacts with Asp105 and Asp141.

Closer inspection of the KAT1 VSD reveals that the length of the S4 helix of KAT1 is comparable to those of Eag1, Kv1.2-2.1, but much shorter than that of HCN1 (Fig. 1g). Furthermore, structural comparison reveals that the S4 helices of Kv1.2-2.1 and Eag1 extend into the extracellular side of the membrane, with the 3_10_-helices located in the center of the membrane, while KAT1 and HCN1 have their S4 helices extending into the cytoplasm side of the membrane (Fig. 1h). We also found that the positive charges of the depolarization-gated channels Kv1.2-2.1 and Eag1 are mostly distributed in the upper part of the S4 helix, while the positive charges of KAT1 are mostly distributed in the lower part of the S4 helix. More interestingly, as a hyperpolarization-gated channel, HCN1 is also active in depolarization.^9^ The extraordinarily long S4 helix of HCN1 extends into both the extracellular and cytoplasmic sides of the membrane, and positive charges are distributed in both the upper and lower parts of the S4 helix (Fig. 1h).

In KAT1, the short, non-helical S4–S5 linker contacts the C-linker of a neighboring subunit through electrostatic and hydrophobic interactions (Fig. 1i). Ile189 in the S4–S5 linker inserts into a hydrophobic pocket formed by Phe309–Val313–Leu334–Cys338 in the C-linker. Moreover, the main-chain oxygen atoms of Asp188 and Phe191 form salt bridges with the side chain of Arg310. The C-terminus of S4 and the N-terminus of S5 form hydrophobic interactions through residues Phe182 and Leu185 in S4 helix, Phe190 in the S4–S5 linker, Ile196 and Lys200 in S5 helix (Fig. 1i). These observations indicate that the S4–S5 linker is the key motif that mediates the coupling between the VSD and the pore of KAT1. We found that mutations of residues located in the S4–S5 linker, Ile189Ala and Arg310Ala, disrupt channel opening, while the Ile189Val mutation leads to a decreased current from KAT1 upon hyperpolarization (Fig. 1j). The interaction between the S4–S5 linker and the C-linker is also observed in the HCN1 channel, where it was mediated by polar interactions.^5^ It was reported that mutation of several specific residues located in the S4–S5 linker of mouse HCN2 channels disrupted channel closure.^13^ Interestingly, the S4–S5 linkers of the depolarization-gated channels Eag1 and hERG have no contact with the C-linkers. These observations suggest that the interaction between the S4–S5 linker and the C-linker may play a critical role in the gating of hyperpolarization-gated ion channels.

Based on the structure, we propose a model for voltage-dependent gating of the KAT1 channel (Supplementary information, Fig. S9). At potentials near 0 mV, the gate is stabilized in a closed conformation through the contact between the S4–S5 linker and the C-linker. This contact can be made in the depolarized conformation of the VSD because S4 has a C-terminal extension following the positively charged region. When the membrane is hyperpolarized, the electric field across the membrane pushes the S4 helix to move inward, resulting in twisting of the attached S4–S5 linker. The stable interaction between the S4–S5 linker and C-linker in turn permits the C-linker to adopt the correct orientation and twist the C-linker disk. The conformational changes in C-linker could wrap the S6 helix bundle. S6 helices twist apart and increase the aperture of the inner pore region (“opening” the gate).

In summary, we report here the cryo-EM structure of KAT1, a hyperpolarization-gated, inwardly rectifying potassium channel in plants. The structure reveals that KAT1 contains a typical K^+^ selectivity filter and a short non-helical S4–S5 linker that contributes to a non-domain-swapped topology in each subunit. The voltage-sensitive S4 helix, with the positively charged residues mainly distributed in the lower part of the helix, is considered to contribute to the hyperpolarization gating of the channel. The S4–S5 linker and the C-linker of the neighbouring subunit form a stable contact, mediating the coupling between the VSD and the pore. The coupled S4–S5 linker and C-linker is proposed to transmit the inward movement of S4 helix under hyperpolarization to the pore opening of the KAT1 channel. Recently, structural studies and molecular dynamics simulations suggested that the significant downward movement of S4 and the subsequent bending of S4 control the opening of the HCN1 channel.^13–15^ Structural and functional studies of KAT1, as well as those of KAT1em,^8^ highlight the divergence between the regulatory mechanism of HCN channels and the direct coupling mechanism suggested here for KAT1. Therefore, this structure greatly advances our knowledge of the gating properties of hyperpolarization-activated ion channels and serves as a guide for structural/functional analyses of plant potassium channels.

## Supplementary information

Supplementary information

## Data Availability

The cryo-EM density map and corresponding coordinate have been deposited in the Protein Data Bank (http://www.rcsb.org/pdb) with code 7CAL, and EMDB (http://www.ebi.ac.uk/pdbe/emdb/) with code EMD-30334.
