# Supplementary material for: Cryo-EM structure of the hyperpolarization-activated inwardly rectifying potassium channel KAT1 from Arabidopsis
Source: Cell Res. 2020 Sep 8;30(11):1049–52. doi: 10.1038/s41422-020-00407-3 (PMC7784887; doi:10.1038/s41422-020-00407-3)
Supplement: Supplementary file 1 — Supplementary information [file 41422_2020_407_MOESM1_ESM.pdf]

Supplementary information

Methods and Meterials

Protein expression and purification

The optimized coding DNAs for *Arabidopsis thaliana* KAT1 (Uniprot: Q39128) and KAB1 (Uniprot: O23016) were synthesized by Sangon Biotech (Shanghai). The KAT1 and KAB1 were cloned into pFastBac-Dual vector with FLAG tag at the amino terminus of KAT1. Baculovirus-infected *Sf9* cells (Thermo Fisher) were used for overexpression and were grown at 27 ℃ in serum-free SIM SF medium (Sino Biological Inc.). Cells were harvested 60 h after infection by centrifugation at 1,000 ×g.

KAT1 protein was purified at 4 ℃. The cell pellet from 1 L culture was resuspended in extraction buffer (10 mM lauryl maltose neopentyl glycol (LMNG), 2 mM cholesteryl hemisuccinate (CHS), 300 mM KCl, 20 mM Tris pH 8.0 and protease inhibitor cocktail (Roche)) for 1.5 hr. Solubilized membranes were clarified by centrifugation at 20000 × g for 30 min. The supernatant was applied to anti-Flag M2 affinity gel (Sigma) by gravity at 4 ℃. The resin was rinsed four times with the wash buffer (0.02% GDN, 300 mM KCl and 20 mM Tris pH 8.0). The target proteins were eluted with wash buffer plus 200 μg/ml FLAG peptide. The eluent was concentrated by Amicon Ultra centrifugal filter (MWCO 100 kDa), and then injected to a Superose 6 increase column (GE Healthcare) equilibrated with SEC buffer (0.02% GDN, 150 mM KCl, 20 mM Tris pH 8.0 and 2 mM DTT). Peak fractions were pooled and concentrated to 4 mg/ml.

Single-particle cryo-EM data acquisition

Purified KAT1 (3 μl) at a concentration of 4.2 mg/ml was added to the freshly plasma-cleaned holey carbon grid (Quantifoil, R1.2/1.3, 300 mesh, Cu), blotted for 6.5 s at 100% humidity with a Vitrobot Mark IV (ThermoFisher Scientific) and plunge frozen into liquid ethane cooled by liquid nitrogen.

Grids were transferred to a Titan Krios electron microscope (FEI) operated at 300 kV equipped with a Gatan K2 Summit direct detection camera. Images of KAT1 were collected using the automated image acquisition software SerialEM[1] in counting mode with 29,000 × magnification yielding a pixel size of 1.014 Å. The total dose of 57.6 $e^-/Å^2$ was fractionated to 36 frames with 0.2 s per frame. Nominal defocus values ranged from -1.6 to -2.3 μm. The dataset of KAT1 included 1,625 micrographs,

respectively.

Image processing

Dose-fractionated image stacks were subjected to beam-induced motion correction and dose-weighting using UCSF MotionCor2[2]. Contrast transfer function parameters were estimated with Gctf[3]. For particle picking, 1,000 - 2,000 particles were picked manually to generate references for auto-picking. The atuo-picked particles were extracted by four-times downscaling resulting in the pixel size of 4.056 Å and then subjected to reference-free 2D classification in Relion-3.0[4]. 200,008 particles from well-defined 2D averages were selected for 3D classification with a pixel size of 2.028 Å. A 3D initial model de novo from the 2D average particles was generated using stochastic gradient descent (SGD) algorithm in Relion. The 50 Å low-pass filtered initial model was used as a template for 3D classification into four classes. A selected subset of 111,658 particles were used to obtain the final map with a pixel size of 1.014 Å and C4 symmetry imposed in the last round of 3D refinement in Relion. The global resolution of this map was estimated to be 3.22 Å based on the gold-standard Fourier shell correlation (FSC) using the 0.143 criterion. Local resolution was determined using ResMap[5] with unfiltered half-reconstructions as input maps.

Model building

The coordinate of human HCN1 (PDB code 5U6O)[6] was fitted into the 3D EM maps of KAT1 using UCSF Chimera[7]. The sequence of HCN1 were mutated with corresponding residues in KAT1 in Coot[8]. Every residue was manually examined. The chemical properties of amino acids were considered during model building. The N-terminal residues 1-48 and C-terminal residues 493-677 were not bulit due to the lack of corresponding densities. Structure refinement and model validation were performed using phenix.real_space_refine module in PHENIX[9,10]. The final model was subjected to refinement and validation in PHENIX.

Plasmid construction, cell culture and transient transfection of HEK293 cells

The coding sequence for wild-type KAT1 was sub-cloned into the pcDNA3.1/Zeo (+) vector. All site-directed mutations were generated with overlap PCR and inserted into pcDNA3.1/Zeo (+). The mutants were sequenced to verify that no unwanted mutations had been introduced. HEK-293T cells,

cultured in DMEM (GIBCO) with 10% FBS (GIBCO), 1% Pen Strep Glutamine (GIBCO) and 0.1% Plasmocin prophylactic (Invitrogen) at 37 ℃ in a 5% CO_2 incubator. The HEK-293T cells were transferred to 24-well plates for transfection. When the cells reached 90% confluence, they were transfected with 0.8 μg of plasmid encoding EGFP and 1 μg of plasmid encoding wild-type or mutant KAT1 (or with 0.8 μg of plasmid encoding mCherry and 1 μg of plasmid encoding wild-type or mutant KAT1 pcDNA3.1-EGFP) using lipofectamine 3000 reagent (Invitrogen). After incubation for 5 h, the cells were transferred to poly-L-lysine (Sigma)-coated slides for culture for another 24-48 h in fresh medium. They were then used for the electrophysiological analysis.

Electrophysiological analysis of HEK293 cells

For the whole-cell recordings, the bath solution contained 150 mM NaCl, 4 mM KCl, 2 mM $CaCl_2$, 1 mM $MgCl_2$, and 10 mM HEPES (pH 7.4, ~308 mOsm). The electrodes were pulled from thick-walled borosilicate glass capillaries with filaments (1.5 mm diameter; Sutter Instruments) on a four-stage puller (P-1000; Sutter, USA) and had resistances of 3-5 MΩ when filled with intracellular solution containing 140 mM KCl, 10 mM NaCl, 5 mM EGTA, 10 mM HEPES pH 7.4, ~297 mOsm). All chemicals were obtained from Sigma. The experiments were performed at room temperature with an EPC-10 amplifier (HEKA Electronic) with the data acquisition software PatchMaster. Representative current traces evoked in response to 2-s voltage pulses from -20 to -180 mV in -20 mV increments from a holding voltage of -80 mV. Tail currents were recorded at -100 mV. The pulses were applied every 20 s. Tail current values were normalized to compare current–voltage relationships between the wild-type and mutants.

References:

1 Mastronarde, D. N. Automated electron microscope tomography using robust prediction of specimen movements. *J Struct Biol* **152**, 36-51(2005).

2 Zheng, S. Q. *et al.* MotionCor2: anisotropic correction of beam-induced motion for improved cryo-electron microscopy. *Nat Methods* **14**, 331-332 (2017).

3 Zhang, K. Gctf: Real-time CTF determination and correction. *J Struct Biol* **193**, 1-12(2016).

4 Zivanov, J. *et al.* New tools for automated high-resolution cryo-EM structure determination in

RELION-3. *Elife* **7**, e42166(2018).

5 Kucukelbir, A., Sigworth, F. J. & Tagare, H. D. Quantifying the local resolution of cryo-EM density maps. *Nat Methods* **11**, 63-65(2014).

6 Lee, C.-H. & MacKinnon, R. Structures of the Human HCN1 Hyperpolarization-Activated Channel. *Cell* **168**, 111-120.e111(2017).

7 Pettersen, E. F. *et al.* UCSF Chimera--a visualization system for exploratory research and analysis. *J Comput Chem* **25**, 1605-1612(2004).

8 Emsley, P., Lohkamp, B., Scott, W. G. & Cowtan, K. Features and development of Coot. *Acta Crystallogr D Biol Crystallogr* **66**, 486-501(2010).

9 Afonine, P. V. *et al.* New tools for the analysis and validation of cryo-EM maps and atomic models. *Acta Crystallogr D Struct Biol* **74**, 814-840(2018).

10 Adams, P. D. *et al.* PHENIX: a comprehensive Python-based system for macromolecular structure solution. *Acta Crystallogr D Biol Crystallogr* **66**, 213-221(2010).

Supplementary information, Figures

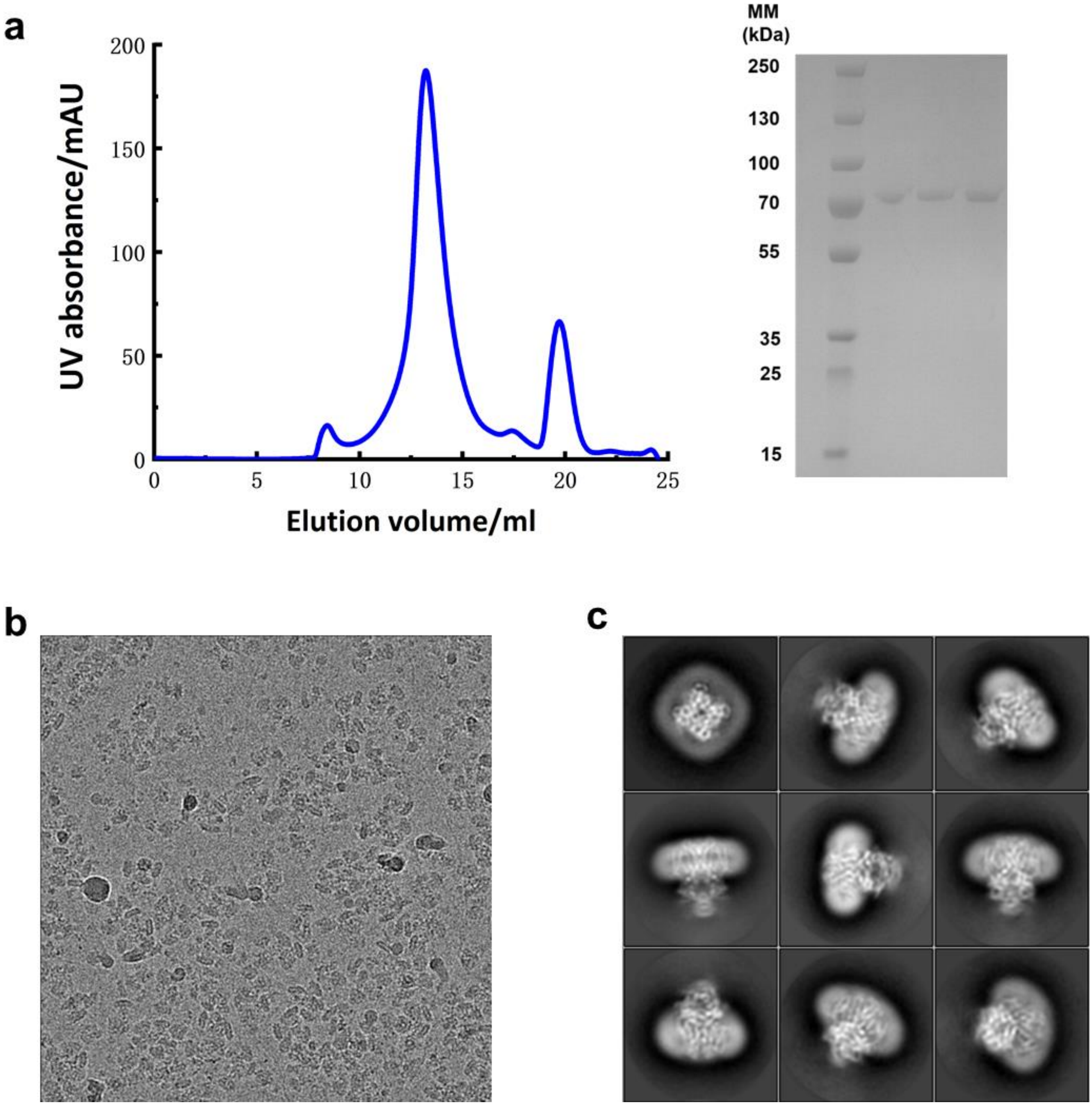

Fig. S1. Purification and cryo-EM sample preparation of KAT1. **a** Size-exclusion chromatography (left) and SDS-PAGE analysis (right) for purification. **b** and **c** A representative cryo-EM micrograph (b) and Representative 2D class averages (c) of KAT1.

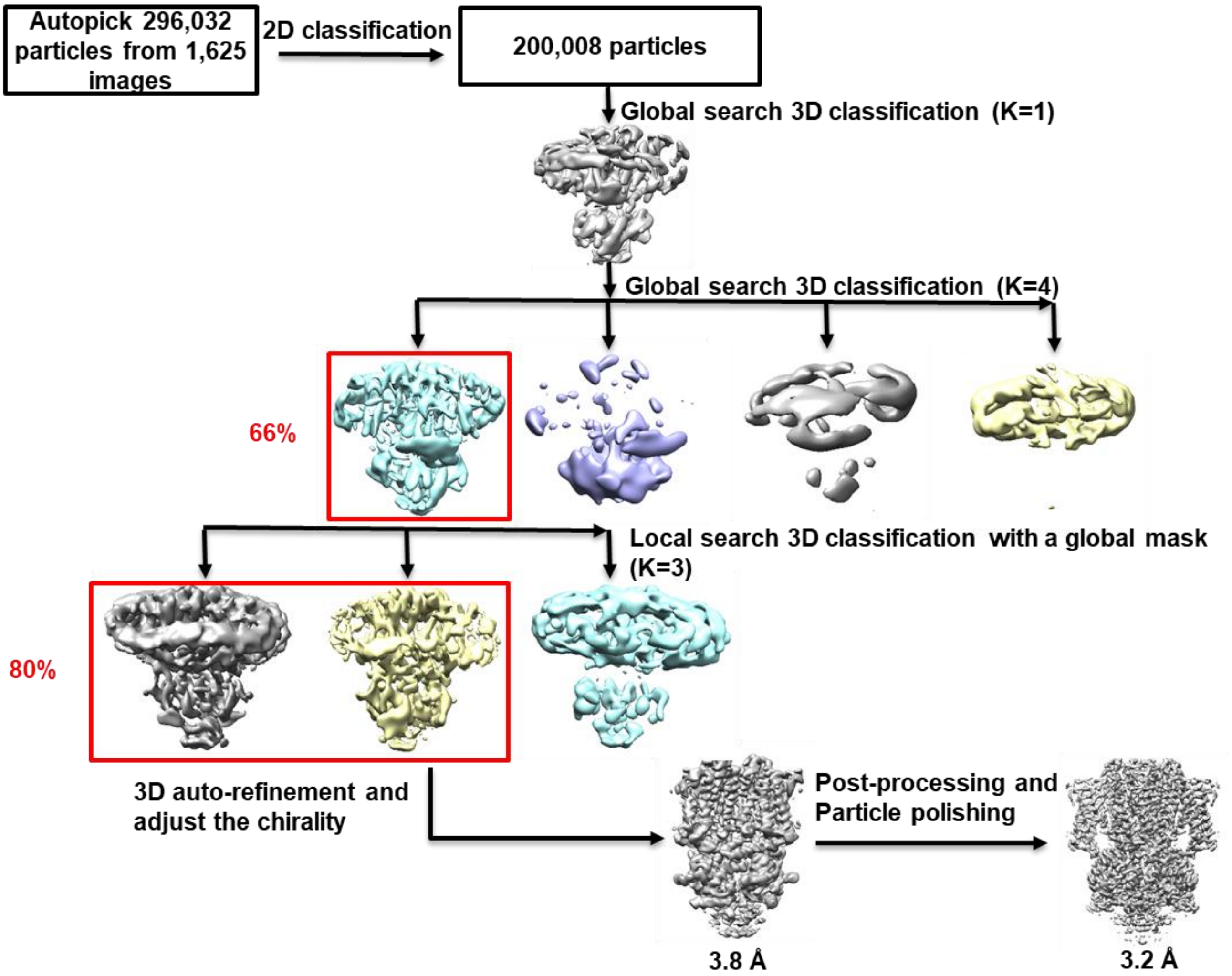

Fig. S2. A flowchart of the cryo-EM data processing and structure determination for KAT1.

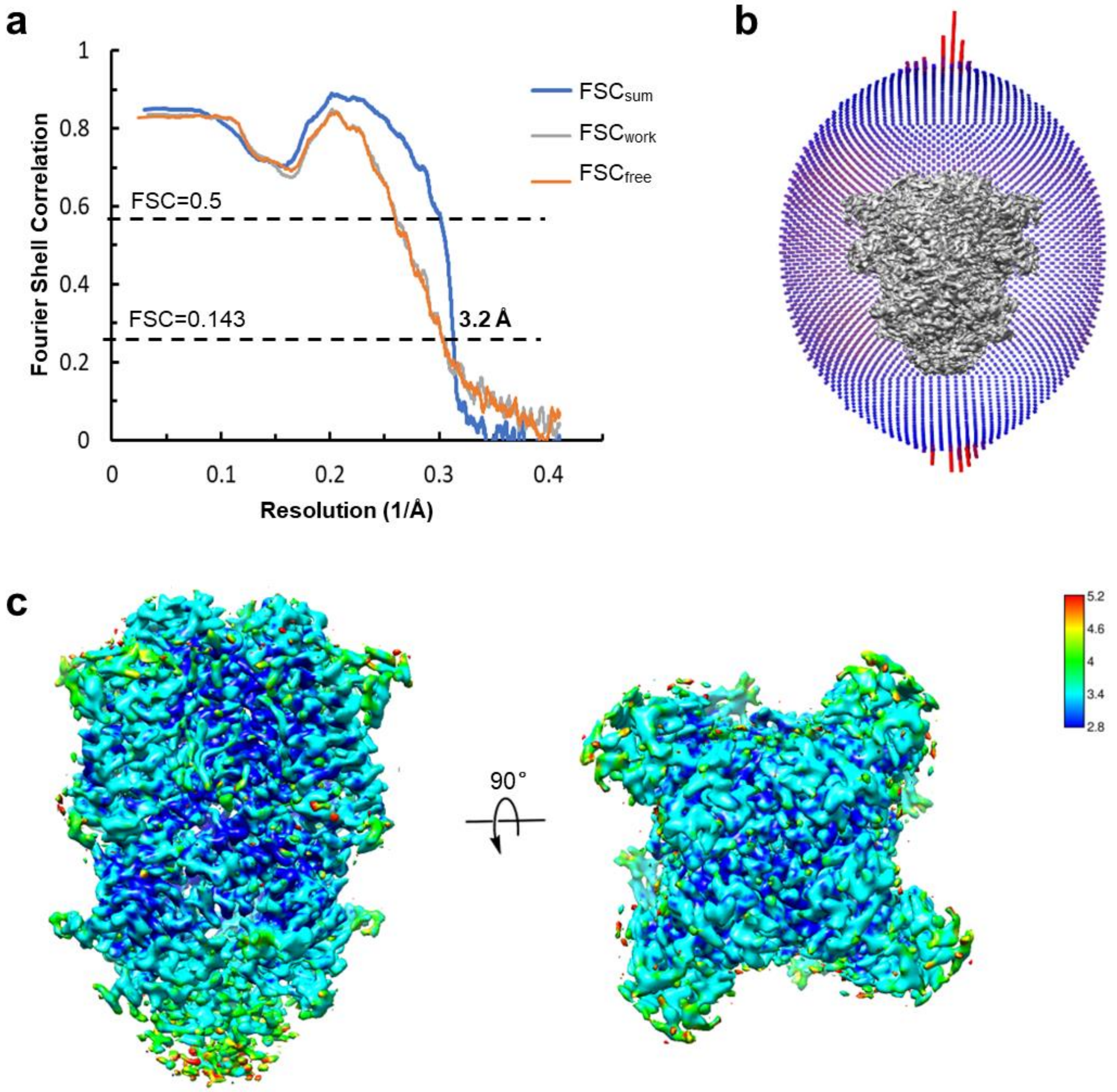

Fig. S3. Cryo-EM structure determination and validation of KAT1. **a** Gold-standard Fourier shell correlation (FSC) curves for the 3D EM reconstruction of KAT1. FSC_{sum} (blue) is calculated for the full masked map and the model refined against the complete dataset; FSC_{work} (grey) is calculated for the masked first half-map and the shaken model refined against the dataset comprising the first half map; FSC^{free} (orange) is calculated for the masked second half-map and the shaken model refined against the first half map. FSC thresholds at 0.5 and 0.143 were used for FSC_{sum} and FSC_{work}/FSC_{free}, respectively. **b** Particle angular distribution of the final cryo-EM reconstruction of KAT1. **c** Local resolution map of KAT1. The map was estimated with RELION 3.0 and generated in Chimera.

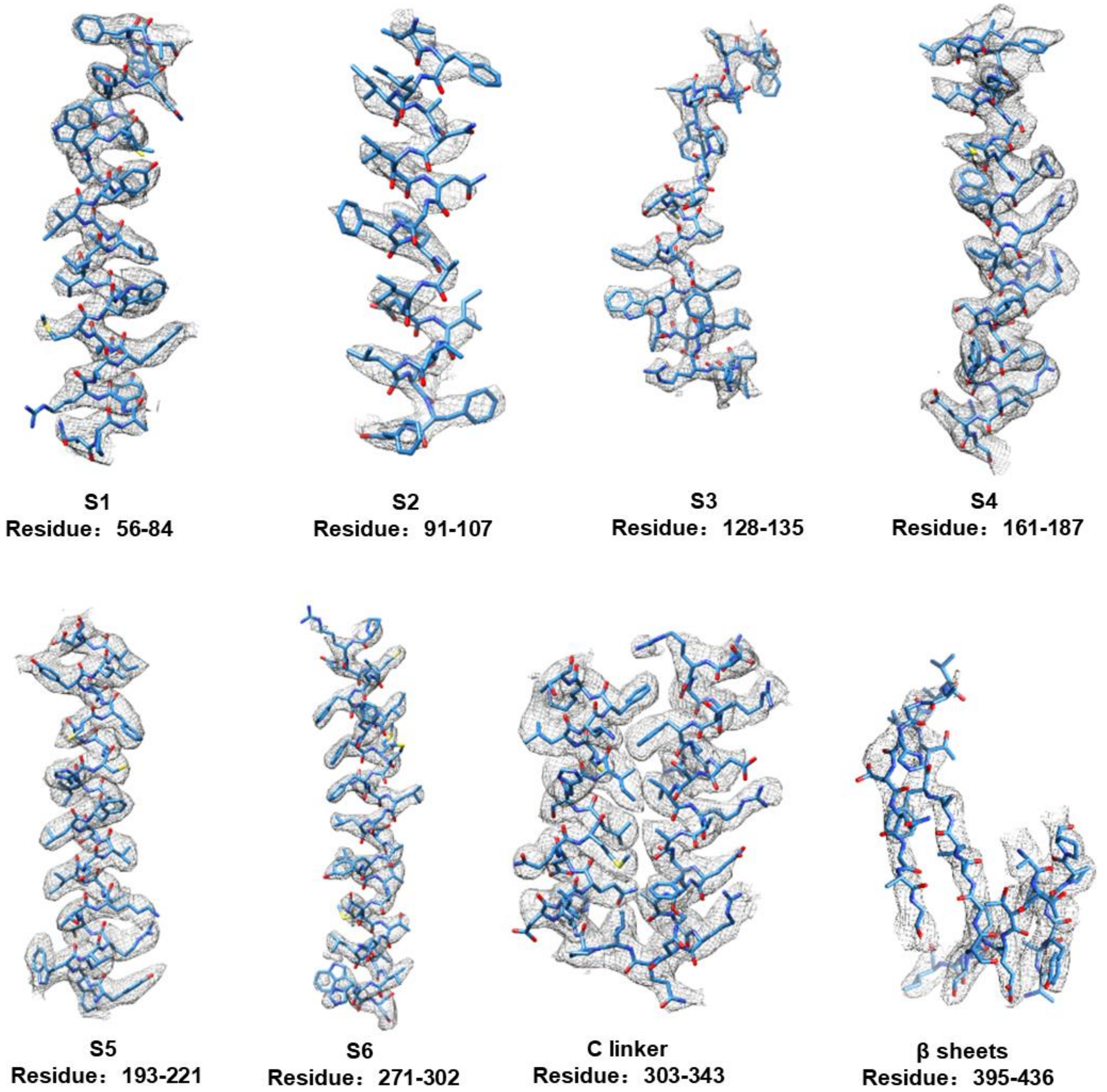

Fig. S4. Agreement between the cryo-EM map and the model of KAT1. Representative cryo-EM densities and fitted atomic models for KAT1.

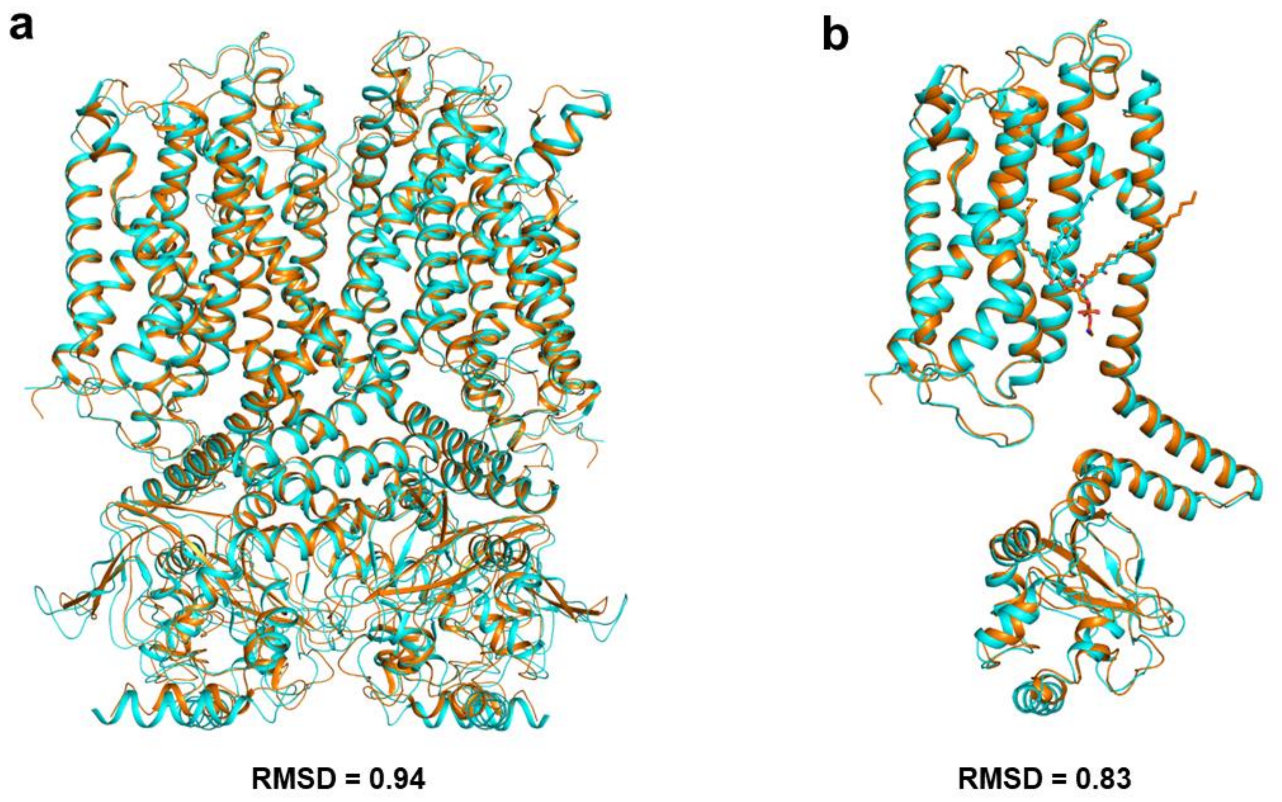

Fig. S5. Structure comparison of KAT1 and KAT1em. **a** Overlay of KAT1 (colored orange) and KAT1em (colored cyan, PDB: 6V1X), viewed from the extracellular side. **b** Overlay of a single subunit of KAT1 (colored orange) and KAT1em (colored cyan, , PDB: 6V1X).

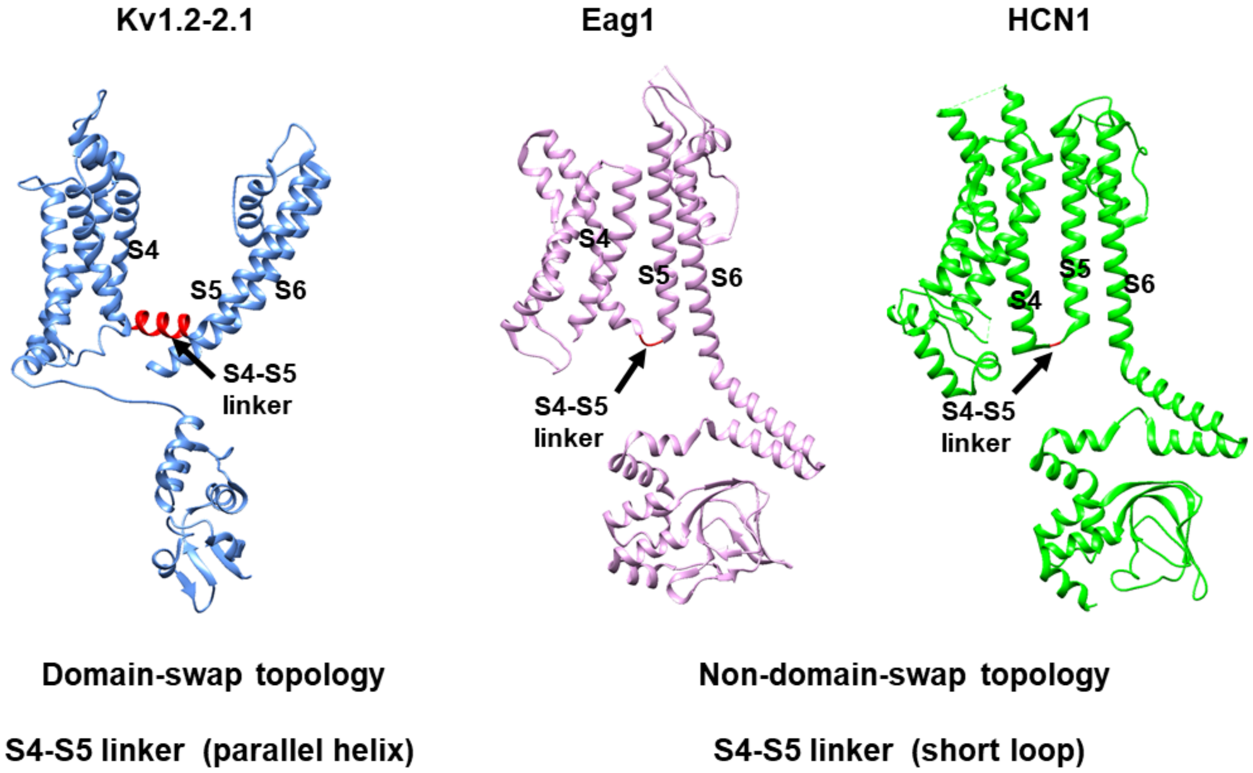

Fig. S6. Structural comparison of the S4-S5 linkers of Domain-swapped potassium channel and Non-domain-swapped ion channels. Overview structures of a single subunit of Kv1.2-2.1 (PDB: 2R9R, slate), Eag1 (PDB: 5K7L, pink) and HCN1 (PDB: 5U6O, green). S4-S5 likers are colored red.

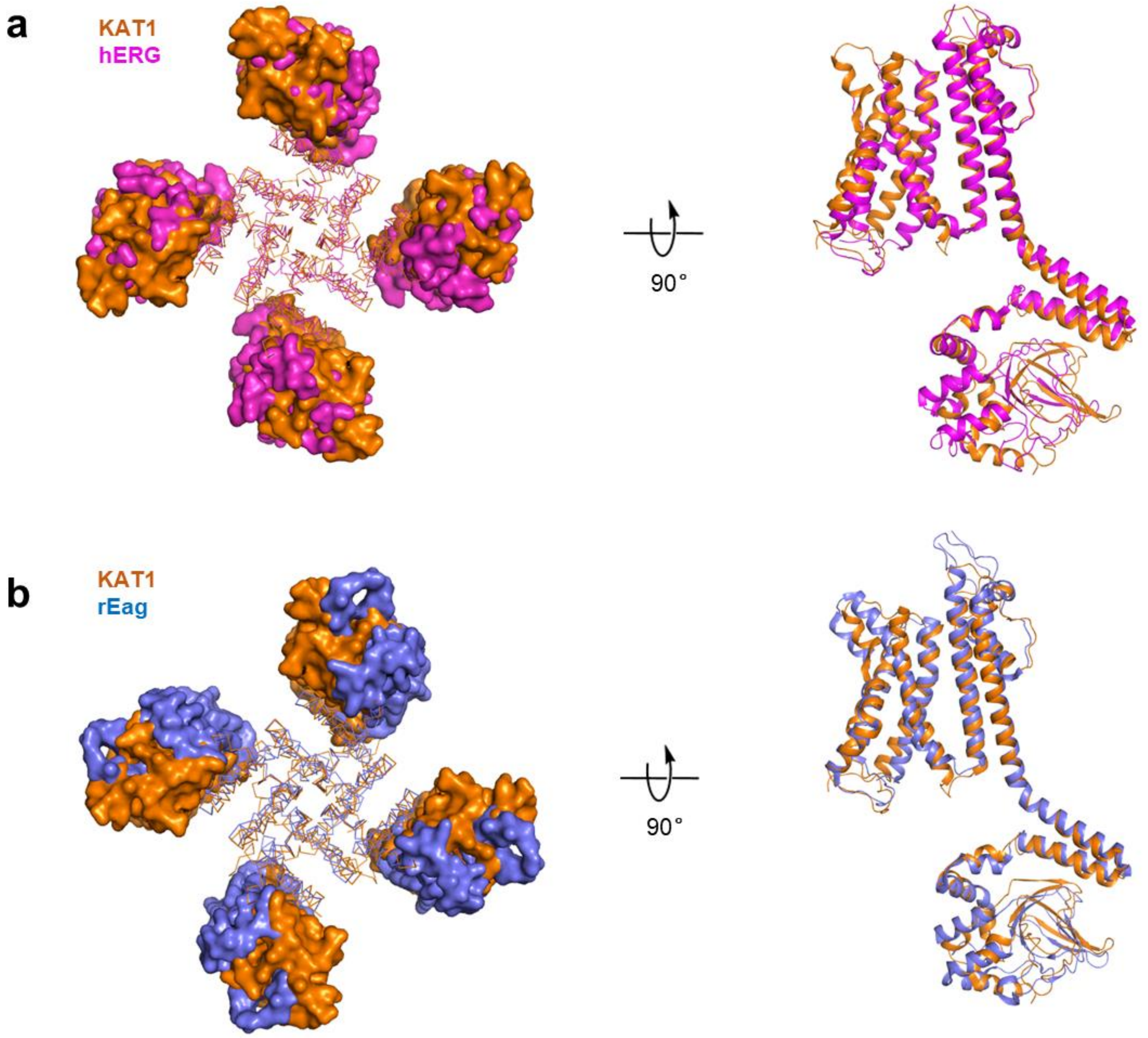

Fig. S7. KAT1 adopts non-domain-swapped structure. **a** and **b** (left panel) Overlay of KAT1 (colored orange) and hERG (a, colored magenta, PDB: 5VA1), Eag1 (b, colored blue, PDB: 5K7L), viewed along the 4-fold axis, from the extracellular side. The two channels are aligned with respect to the pore domain (shown as Ca traces) and the VSDs are shown as surfaces. Only the transmembrane region is shown for clarity. **a** and **b** (right panel) Overlay of a single subunit of KAT1 (colored orange) and hERG (a, colored magenta, PDB: 5VA), Eag1 (b, colored blue, PDB: 5K7L).

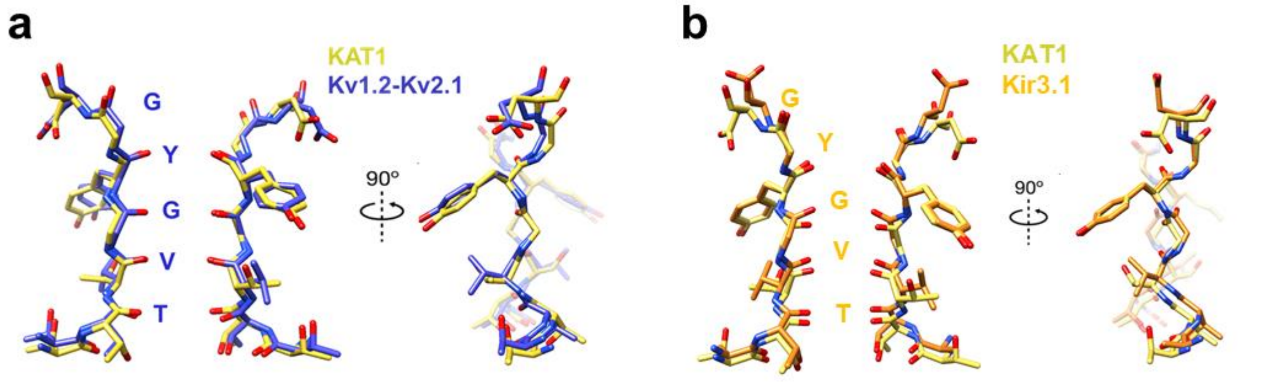

Fig. S8. Structure comparison of the filters from KAT1 and Kv1.2-Kv2.1 (a, PDB: 2R9R), Kir3.1 (b, PDB: 2QKS)

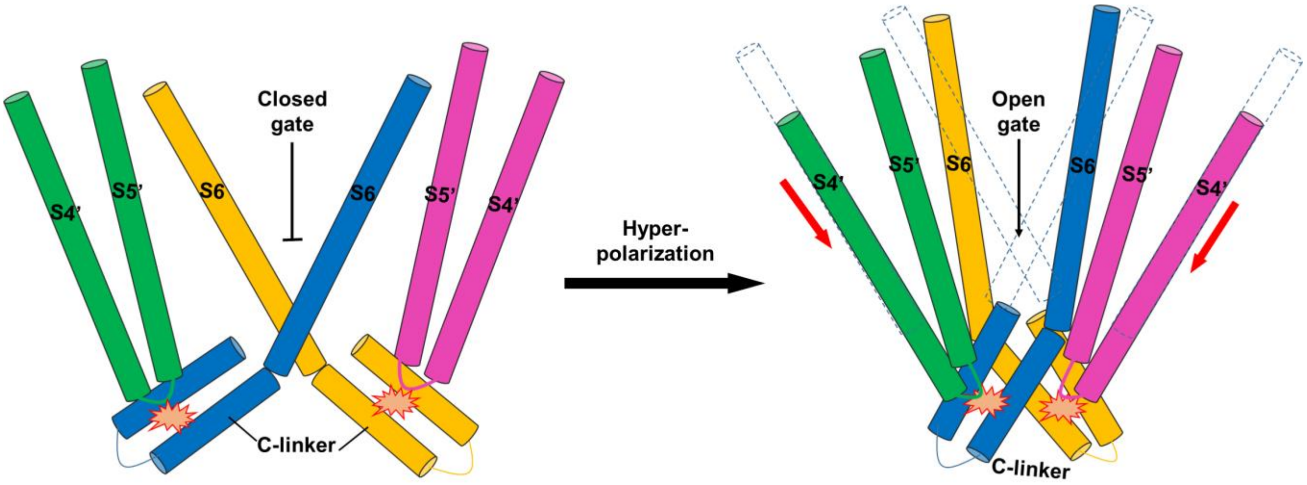

Fig. S9. Proposed mechanism of hyperpolarization gating of the KAT1 channel. Diagrams of closed (left) and open (right) ion channels shown through the plane of the membrane. VSD helices S1-S3 of the four subunits have been removed for clarity. Helices divided from the four subunits are shown as cylinders in different colours. The stable interaction between the S4-S5 linker and the C-linker from an adjacent subunit transmits the inward movement of S4 helix under hyperpolarization to the S6 helix, twisting the S6 helix and opening the gate.

Supplementary information, Table S1

Statistics of cryo-EM data collection, 3D reconstruction and model refinement.

**Data Collection**	
Protein	KAT1
Microscope	FEI Titan Krios
Voltage (kV)	300
Detector	Gatan K2 Summit
Detector mode	Counting
Pixel size (Å)	1.014
Defocus range (μm)	-1.6 ~ -2.3
Electron dose (e^-/Å^2)	57.6
Frames per image	36
Exposure time (s)	8
Number of images	1,625
**3D reconstruction**	
Software	Relion 3.0
Particle number	111,685
Symmetry	C4
Overall resolution (Å)	3.2
**Model refinement**	
Software	Phenix
Bonds (RMSD)	
Length (Å)	0.006
Angles (°)	1.007
Ramachandran plot (%)	
Outliers	0.00
Allowed	10.86
Favored	89.14
Rotamer outliers (%)	0.86
MolProbity score	1.75
Clash score	3.58
